# The Metabolome Atlas of 22 Tissues in Aging Mice Reveals a Switch in Thermogenesis from Brown Fat to Skeletal Muscle

**DOI:** 10.21203/rs.3.rs-7811947/v1

**Published:** 2025-10-29

**Authors:** Oliver Fiehn, Min Liu, Jian Ji, Anthony David, Huaxu Yu, Tong Shen, Yuanyue Li, Lydia Yang

**Affiliations:** University of California, Davis; University of California, Davis; State Key Laboratory of Food Science and Technology, National Engineering Research Center for Functional Foods, Synergetic Innovation Center of Food Safety and Nutrition, Jiangnan University; University of California, Davis; University of California, Davis; UC Davis; Department of Hepatobiliary and Pancreatic Surgery, The First Affiliated Hospital, Zhejiang UniversitySchool of Medicine; University of California, Davis

## Abstract

Aging impairs thermoregulatory capacity, yet the metabolic mechanisms remain unclear. We report an organ-resolved metabolome atlas of 2,875 structurally annotated metabolites of old (90–96 weeks) versus young mice (16 weeks) across 22 tissues and four biological matrices. For thermoregulation, aging induces widespread remodeling of mitochondrial cardiolipins, with severe depletion of nascent species in brown adipose tissue (BAT) and a compensatory shift in thermogenic workload from BAT to muscle, evidenced by higher levels of long-chain fatty acids, acylcarnitines, and ω-oxidation markers in quadriceps. BAT showed reduced lipolysis and lower levels of the thermogenic lipokine 12,13-DiHOME, whereas muscle exhibited increased 12,13-DiHOME, lipid uptake, β-oxidation, and stress-associated metabolites including oxidized/reduced glutathione ratio. Hence, thermogenic adaptation comes at a cost: aged muscles exhibited signs of proteostatic stress, energetic strain, and oxidative damage, suggesting compensation contributes to sarcopenia. The atlas is publicly available at GitHub https://github.com/minliuUCDavis/AgingMiceAtlas and serves as a cornerstone resource for aging biology.

## Introduction

Aging is a complex, multifactorial process marked by a progressive physiological decline^1^. Older adults face a disproportionately high burden of heat- and cold-related morbidity and mortality^1, 2, 3, 4^. Epidemiological analyses revealed that most deaths attributable to hypo- or hyperthermia occur in the elderly^2, 3, 4^. Even within non-extreme ambient ranges, mild cooling elicits larger falls in core body temperature in older than young adults, consistent with age-related deficits in autonomic thermoregulation^5, 6^. Globally, failure to adapt to environmental temperature changes account for over 5 million deaths annually (2000–2019), with the burden attributable to cold approximately ninefold greater than that attributable to heat^7^. Although aging reshapes metabolism throughout the body, the organ-specific sources of thermoregulatory failure remain poorly defined, including specifics how age-related impairments in temperature sensation, heat conservation, heat dissipation, endogenous thermogenesis, and physiological tolerance^8^ interact to heighten risk of death in late life, particularly at the level of specific organs.

Tissues central to adaptive thermogenesis, particularly brown adipose tissue (BAT) and skeletal muscle, warrant focused attention^9^. Both undergo age-related atrophy and functional decline^10, 11^, yet their relative and coordinated contributions to thermoregulatory failure remain unclear. BAT and skeletal muscle are mitochondria-dense and mitochondria-dependent tissues for thermogenesis. However, the specific mitochondrial biochemical changes that occur with age in these tissues, and how such changes impair adaptive thermogenesis, are not known. Similarly, it is unclear how the body orchestrates intertissue crosstalk between thermogenic organs during aging, and whether their collective degradation is a key driver of the thermoregulatory deficits. Understanding the metabolic rewiring within and between these organs may reveal mechanisms underlying the elevated risk of hypothermia in the elderly.

Here, we present an organ-resolved metabolome atlas of aging in mice spanning 22 tissues and four associated biological matrices, comparing old (90–96 weeks) with young (16 weeks) adult animals. We annotated 2,875 unique metabolites across major biochemical classes, providing a resource to localize the tissue origins of age-related metabolic change. We examine inner mitochondrial membrane (IMM) lipid remodeling as surrogate for mitochondrial function and thermogenesis. We investigate thermogenesis-relevant biochemical changes across tissues, including for lipid-mediator signaling that influence thermogenic control. Our results indicate that aging entails a systemic recalibration of energy expenditure characterized by a redistribution of thermogenic workload from BAT toward skeletal muscle. By using thermogenesis as an example, we provide a framework for further mechanistic studies. The organ-resolved aging metabolome atlas is openly available on GitHub (https://github.com/minliuUCDavis/AgingMiceAtlas), with an interactive browser to be released for easy public access.

## Results

### A multi-organ metabolome atlas of aging mice

**Figure 1:**
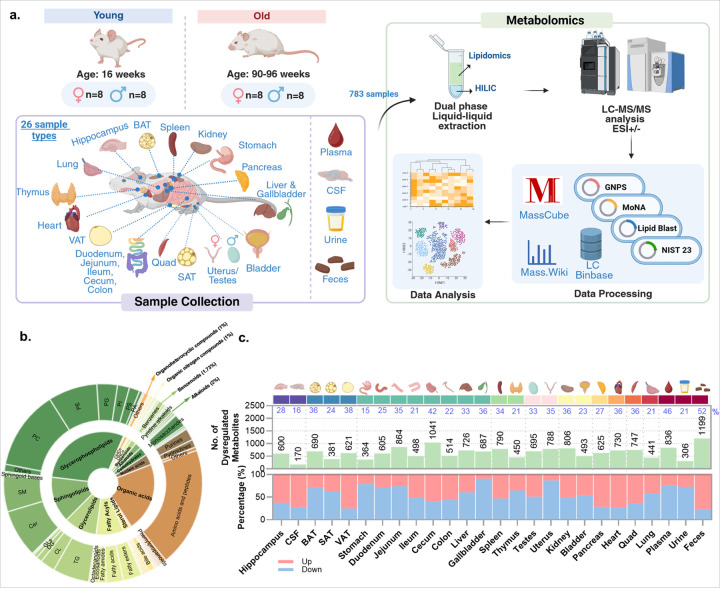
An organ-resolved metabolome atlas of aging mice. **(a)** Study design and analytical workflow. **(b)** Chemical composition of the annotated metabolites according to RefMet classification. **(c)** Organ-resolved aging signatures. **Upper panel:** per-tissue counts of metabolites with raw p < 0.05 (old vs young; two-sided Mann–Whitney U). Blue percentage labels indicate dysregulated/total detected per tissue. **Lower panel:** fraction of increases vs decreases.

We present an organ-resolved atlas of metabolism in 90–96 weeks old mice versus 16 weeks young mice (n=8 males and n=8 females per age, all C57BL/6NCrl genetic background). Immediately after euthanasia we collected 22 tissues plus plasma, cerebrospinal fluid (CSF), urine and feces, yielding 783 samples in total. Metabolite data were acquired by hydrophilic interaction chromatography (HILIC) for polar metabolites and reversed phase (RP) liquid chromatography (LC) for complex lipids (Fig. 1a) with high resolution mass spectrometry in positive and negative electrospray. 2,875 unique compounds were annotated by MS/MS spectral matching against spectral libraries yielded annotations spanning 18 RefMet superclasses^12^, capturing broad chemical diversity including sterol lipids, fatty acyls, glycerophospholipids, nucleotides, amino acids and peptides (Fig.1b). All annotations are publicly available at Mass.Wiki to invite scientific review and comments of the data, and only annotated metabolites were used in statistical and functional analyses. Across all samples, the specific organ or tissue identity dominated the variance in the dataset. UMAP projections separated nearly every tissue and biological matrices into distinct clusters, whereas age and sex exerted smaller effects (**Extended Data Fig.1a, b & c**). To summarize aging effects per organ, we compared old versus young using two-sided Mann-Whitney U tests and counted dysregulated metabolites at raw *p* <0.05, enabling overall biochemical phenotyping given the large number of metabolites assayed (Fig.1c). For comparison, statistical data are given in **Supplementary Table 1**, including FDR corrections. Overall, the highest proportions of age-differentiated metabolites were observed in feces (52%), plasma (46%), cecum (42%), and visceral adipose tissue (VAT; 38%). The directionality of age-based regulation differed by the physiological system: the hippocampus, CSF, pancreas, heart and quadriceps were enriched for metabolites that showed increased levels with age, whereas the stomach, duodenum, jejunum, liver and gallbladder were dominated by down-regulated metabolites. Notably, 76% of altered metabolites in feces showed increased levels. Adipose depots diverged: 70% of age-altered metabolites decreased in brown adipose tissue (BAT), while 75% of the metabolites increased in VAT, indicating a vigorous remodeling of adipose metabolism during aging. We also found sex–age interactions (two-way ANOVA, FDR <0.05) to be tissue-specific, with up to 50% of age-dysregulated metabolites showing sex dependence in some organs but not in others (**Extended Data Fig.1d**). Lipid classes such as sphingolipids, ether glycerophospholipids and glycerophospholipids contributed disproportionately to these interactions.

### System-wide dysregulation of cardiolipins across aging organs

**Figure 2:**
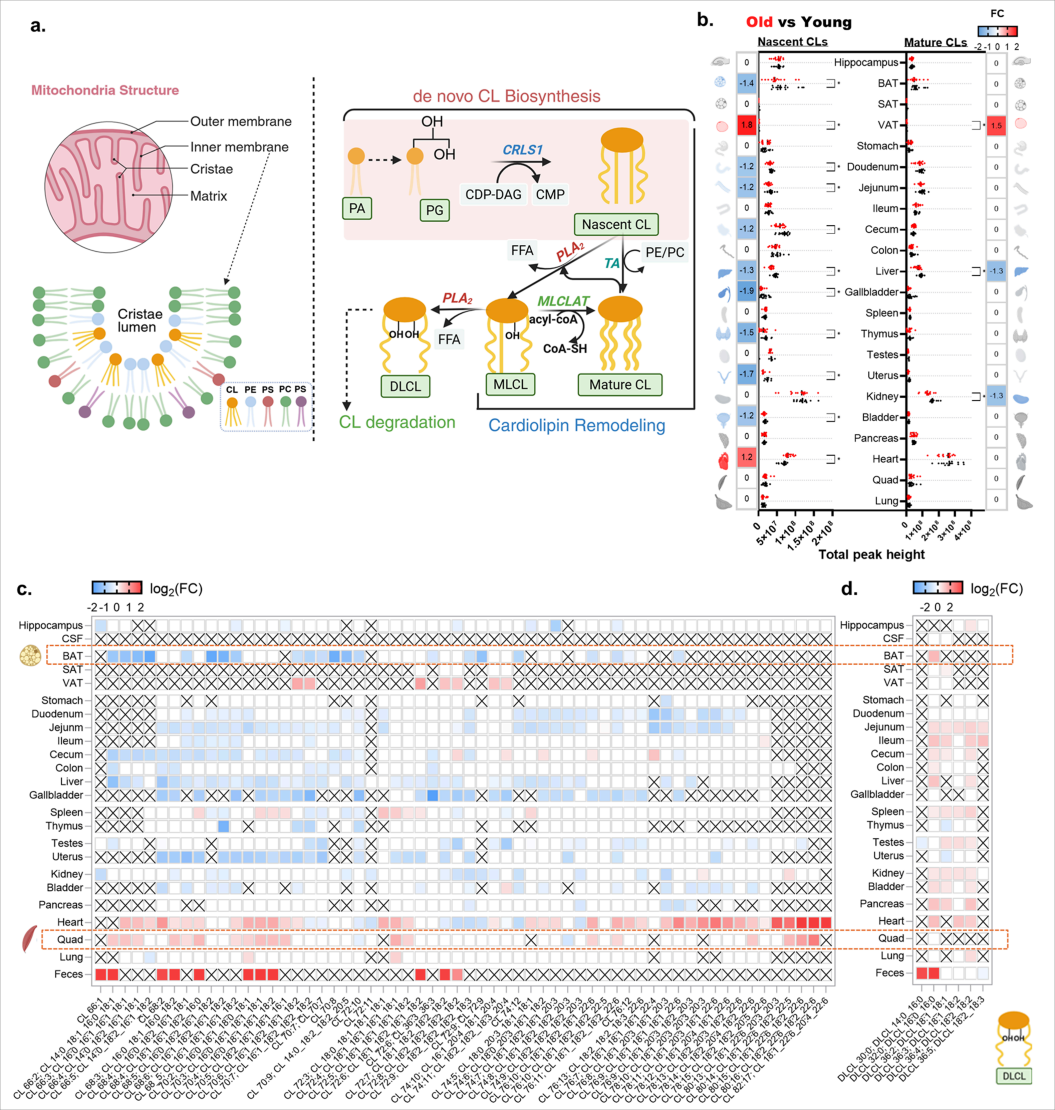
System-wide remodeling of cardiolipins in aging. **(a)** Schematic of the inner mitochondrial membrane (IMM), highlighting cristae and the cardiolipin (CL) pathway of de novo synthesis and remodeling/diacylation-reacylation (adapted from Ref.^18^). Abbreviations: PA, phosphatidic acid; PG, phosphatidylglycerol; CDP-DAG, cytidine diphosphate diacylglycerol; CMP, cytidine monophosphate; Crls1, cardiolipin synthase 1; CL, cardiolipin; TA, Tafazzin; PLA2, phospholipase A2; FFA, free fatty acids; MLCL AT-1, monolyso cardiolipin acyltransferase; MLCL, monolyso cardiolipin; DLCL, dilyso cardiolipin. **(b)** Total sum of nascent and mature CL levels in young and old mice (dots, individual animals; n=16 per group, sex-balanced). Heat maps show fold change (old/young) for nascent (left panel) and mature (right panel) CL pools with a shared diverging color scale (−2 to +2; blue, decrease; red, increase; white, not significant). **(c) & (d)** Species-resolved heat maps of log_2_-fold changes (FC) between old and young mice of CL (c) and DLCL (d) across organs. Shared color scale as in (b); **×**, not detected. Two-sided Mann–Whitney U tests; significance threshold raw *p* <0.05.

Cardiolipins (CLs) are distinctive tetra-acyl phospholipids that constitute a major component of the inner mitochondrial membrane (IMM) and are essential for mitochondrial energy production and membrane architecture^13, 14, 15, 16^ (Fig.2a). Across the 22 tissues, we annotated 66 cardiolipin species including six CL-remodeling intermediates, called dilyso cardiolipins (DLCLs). Because cardiolipins transition from nascent (shorter and more saturated acyl chains; substrates for remodeling) to mature (longer and more unsaturated acyl chains) forms during remodeling, we classified cardiolipins into 36 nascent and 24 mature cardiolipins (Fig.2a; detailed classification criteria described in Supplementary Note.1 and Table 2). We observed significant age-related alterations in cardiolipin compositions across most organs. For each tissue, we compared cardiolipin compositions for old versus young mice with respect to the total sum of nascent and mature CL levels (Fig.2b, two-sided Mann-Whitney U test with FDR-corrected). Aging produced a widespread but organ-specific depletion of total nascent CLs, with half of all tissues showing statistical differences. The strongest decreases for nascent CLs were observed in BAT and VAT, in the gut (duodenum, jejunum, cecum), and in liver, gallbladder, thymus, uterus and bladder (Fig.2b). In contrast, in the heart, total nascent CLs increased at old age. Changes in total mature CL levels with aging were restricted to decreases in liver and kidney.

Heat maps confirmed the broad downregulation of cardiolipin species with aging in BAT and along the gut (Fig.2c). Heart and quadriceps muscles showed increases for many individual CL species, although the aging heart displayed a selective loss of linoleate-rich mature species **(Extended Fig.2a).** We found a sustained decline for tetralinoleoyl cardiolipin CL(18:2)_4_ with age (FC=−1.6; q-value = 0.0009). Because CL(18:2)_4_ is the predominant cardiolipin species in normal skeletal and cardiac muscle and considered optimal for respiratory-chain function^17^, this pattern indicates a selective loss of functional, linoleate-rich cardiolipins despite preserved or increased CL pools during cardiac aging. We then investigated the cardiolipin intermediates, dilyso cardiolipins. Across organs, including jejunum, ileum, cecum, liver, kidney, bladder, pancreas and heart, several DLCL species increased with age (Fig.2d). As DLCLs are indicative of CL degradation (Fig.2a) and concordant with decreases of mature CLs in liver and kidney, these changes indicated a consistent and multi-organ remodeling of CL metabolism in aging, supporting changes in mitochondrial cristae architecture **(Extended Fig.2b).**

### The BAT metabolome shows diminished mitochondrial thermogenesis at old age

**Figure 3:**
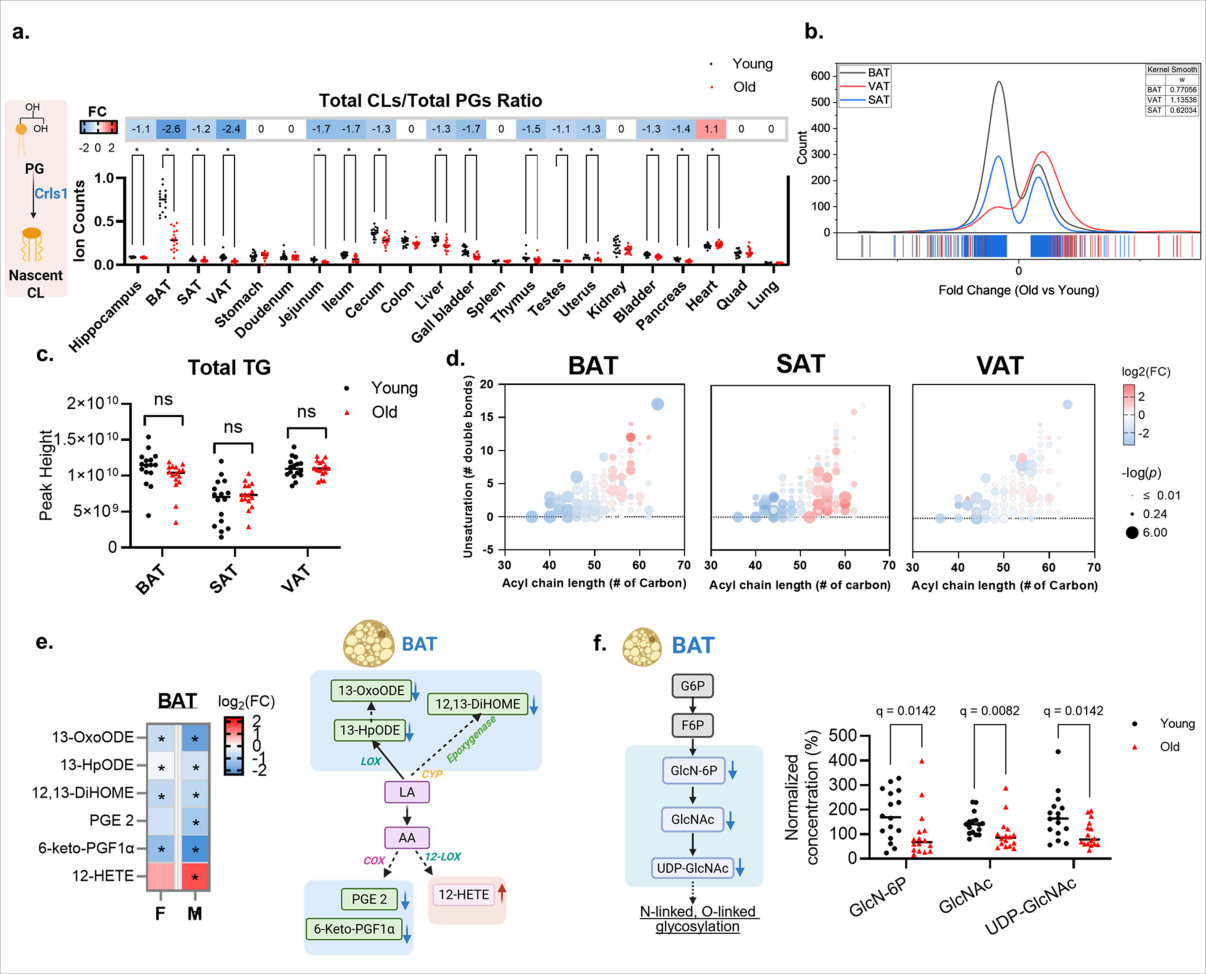
The BAT metabolome shows diminished mitochondrial thermogenesis at old age. **(a)** Proxy for cardiolipin synthesis: ratio of nascent cardiolipins (CLs) to phosphatidylglycerols (PGs) across tissues (dots, individual animals; black denotes young and red denotes old; n=16 per age). Upper panel: heat map of fold change (FC) between old and young mice for the nascent CLs/PG ratio with a shared diverging color scale (−2 to +2; blue, decrease; red, increase; white, not significant). **(b)** Kernel density estimates of fold change (old vs. young) for significantly dysregulated metabolites in BAT (black), VAT (red) and SAT (blue). Positive values denote upregulation; negative values denote down-regulation. Rug ticks mark individual metabolites. The legend reports kernel bandwidth (*w*). **(c)** Total peak heights of triacylglycerols (TGs) in BAT, SAT and VAT for young versus old groups. **(d)** TG species remodeling of BAT, SAT and VAT. Bubble plots position individual TG species by total acyl carbons (x-axis) and number of double bonds (y-axis). Bubble color denotes log_2_ fold change (old vs. young); bubble size denotes −log_10_(*p*). Diverging color scale: blue, decrease; red, increase; white, not significant). **(e)** Left: heat map of log_2_-fold changes (FC) between old and young mice for selected linoleic acid (LA)/arachidonic acid (AA)-derived oxylipins in BAT, stratified by sex (n=8 per sex). Blue, decrease; red, increase; * denotes *p* <0.05). Right: Pathway schematic summarizing age-associated alterations in LA/AA metabolism in BAT, highlighting decreases in 13-HpODE, 13-OxoODE, 12,13-DiHOME, PGE2, and 6-keto-PGF1α, and an increase in 12-HETE. **(f)** Left: Schematic of hexosamine biosynthetic pathway (HBP) in BAT. Right: Normalized concentration (%) of HBP metabolites in young (black) and old (red) mice (n=16 per age). Statistics: two-sided Mann–Whitney U tests; FDR-adjusted q values reported. Abbreviations: LA, linoleic acid; AA, arachidonic acid; G6P, glucose-6-phosphate; F6P, fructose-6-phosphate; GlcN-6P, glucosamine-6-phosphate, GlcNAc, N-acetyl-glucosamine; UDP-GlcNAc, uridine-diphosphate-N-acetyl-glucosamine. Shared color bars are used across heat maps.

To assess the metabolic consequences of cardiolipin dysregulation, we calculated the ratio of product to precursor levels of total nascent CLs to total phosphatidylglycerols (PGs) as a proxy for cardiolipin synthase activity (Crls1). The CL/PG ratio declined at old age across multiple organs and was most reduced in BAT (FC = −2.8; q-value <0.0001, Fig.3a), with a smaller decline in jejunum (FC = −1.7; q-value <0.0001), ileum (FC = −1.7; q-value = 0.0009) and gallbladder (FC = 1.7; q-value = 0.0009). Notably, many PG species increased with age across multiple tissues, particularly those containing higher degree of unsaturation **(Extended Data Fig.3).** Conversely, the CL/PG ratio remained largely unchanged in the heart tissue (FC=1.1; q-value = 0.0483, Fig.3a), showing that the larger negative ratios in BAT and intestinal tract likely reflect reduced activity of Crls1 that is not found in other tissues. Previously, it has been shown that CL supports thermogenic activity in brown and beige fats, specifically through activation of Crls1^19^. Conversely, Crls1 deficiency reduces the activity of UCP1, a thermogenic protein which uncouples metabolic catabolism from ATP generation^19^. To further substantiate this difference in thermogenic processes, we therefore examined metabolic catabolism through overall metabolome trajectories. At the whole-metabolome scale, we found about 863 metabolites that showed statistically significant different levels (at raw *p* <0.05) in BAT. 70% of these compounds were found at decreased levels (Fig.3b), suggesting active catabolism. Conversely, in VAT there were only about 418 compounds with significantly different levels between old and young mice, and 75% of these compounds were found to be increased (Fig.3b), supporting the concept of increased metabolic storage rather than catabolic use. For subcutaneous adipose tissue (SAT), about 495 compounds were differentially regulated at old age, with similar numbers of compounds at increased and at decreased levels (Fig.3b).

Given these contrasting trends in catabolism versus storage, we next assessed the abundance and acyl-chain composition of triacylglycerol (TG) as readouts of fuel storage versus catabolism. Total TG abundance did not change significantly with aging in BAT, SAT or VAT (Fig.3c). By contrast, TG composition shifted by acyl-chain length and unsaturation in an adipocyte-specific manner (Fig.3d). Aging BAT was depleted of short- and medium-chain TGs but showed an increase in long-chain TGs (Fig.3d). This observation is consistent with a reduced availability of rapidly mobilized fuels and remodeling toward more inert droplets^20, 21^. SAT showed a similar depletion together with relative enrichment of longer and more unsaturated species, whereas VAT exhibited minimal species-level change. Thus, aging alters the composition rather than the quantity of TG stores, constraining the availability of readily available thermogenic fat.

Consistent with reduced thermogenic tone, we observed coordinated declines in oxylipins linked to BAT activation and lipid handling, most notably 12,13-dihyroxy-9Z-octadecenoic acid (12,13-DiHOME), 13-hydroperoxyoctadecadienoic acid (13-HpODE), 13-oxo-9E,11E-octadecadienoic acid (13-OxoODE) and prostaglandin E2 (PGE 2, Fig. 3e). PGE2 was found as particularly significant downregulated in males but not in females (Fig.3e). Oxylipins are important and long-established mediators of physiology and metabolism^22^. For example, 12,13-DiHOME activates the import of fatty acids into BAT cells and other tissues, and promotes β-oxidation^23^. Conversely, PGE2 promotes the differentiation of white-to-brown adipogenic cells^24^. Both oxylipins were found decreased at old age in BAT, consistent with diminished thermogenic signaling. Moreover, we found decreased levels of hexosamine biosynthetic pathway metabolites (glucosamine-6-phosphate, N-acetylglucosamine, and uridine 5’-diphosphate-N-acetylglucosamine) which are required for effective O- and N-linked glycosylation, processes that stabilize nutrient transporters and fine-tune mitochondrial oxidative phosphorylation^25^. Hence, the hexosamine-pathway data support the notion that nutrient sensing for fat oxidation was reduced in BAT in old age mice. In combination, these results indicate that aged BAT exhibits less short- and medium chain thermogenic fats, diminished pro-thermogenic signaling via oxylipins and blunted activation of thermogenic oxidation through reduced nutrient sensing.

### Adaptive thermogenic workload shifts from BAT toward skeletal muscle

**Figure 4:**
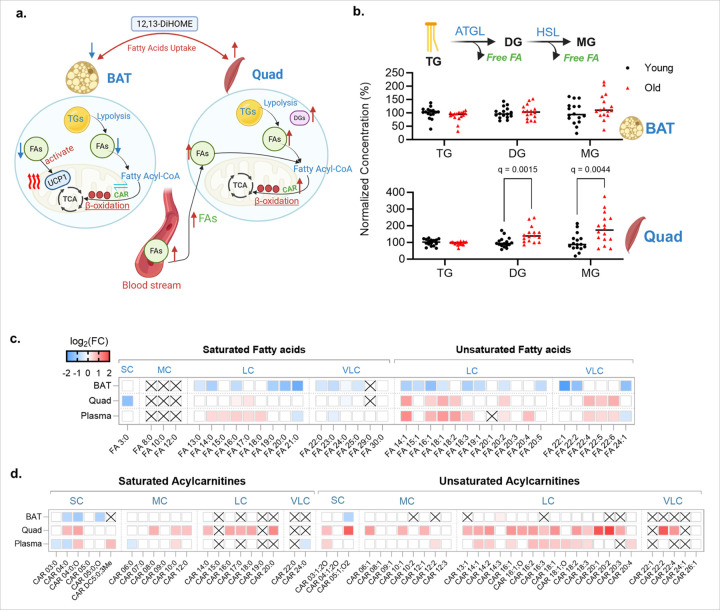
Aging re-partitions lipid oxidation toward skeletal muscle. **(a)** Sketch of the thermogenic switch at old age between BAT and quadriceps muscle. **(b)** Remodeling of neutral lipids in BAT and quadriceps muscle. Bulk triacylglycerol (TG), bulk diacylglycerol (DG) and bulk monoacylglycerol (MG) Dots: individual animals; horizontal lines: median; q values: FDR-adjusted p-values. **(c)** Heat map of log_2_-fold changes (FC) between old and young mice for saturated and unsaturated free fatty acids grouped by short/median/long/very-long chain lengths (SC/MC/LC/VLC). **×** denotes not detected. **(d)** Heat map of log_2_-fold changes (FC) between old and young mice for short/median/long/very-long chain acylcarnitines. **×** denotes not detected.

As non-shivering thermogenesis in BAT declines with age, adaptive thermogenic workload is expected to redistribute across tissues. Correspondingly, we observed coordinated shifts in fuel use across adipose depots and circulation, with quadriceps muscle emerging as a key sink for lipid oxidation (Fig.4a). In quadriceps, bulk triacylglycerol (TG) levels did not increase with age, whereas diacylglycerol (DG) and monoacylglycerol (MG) levels rose, consistent with heightened intramuscular lipolysis. In contrast, bulk TG, DG and MG levels were not significantly different in BAT between young and old-age mice (Fig.4b). Although total TG was unchanged, quadriceps TG composition shifted in old-age mice, in a similar way as in BAT: short- and medium-chain saturated species were depleted and longer, more unsaturated species were relatively enriched **(Extended Data Fig. 4a).** Hence, the compositional remodeling of overall stable bulk TG levels, in combination with DG and MG accumulation, is evidence of increased lipid turnover in muscle tissue of old-age mice. Interestingly, levels of free fatty acids in plasma support this view of multi-organ remodeling of lipid mobilization and use. Plasma from old-age mice showed increases in long-chain (LC) and unsaturated very-long-chain (VLC) fatty acids (Fig.4c). In line with these results, levels of key unesterified (free) long-chain fatty acids (C18:2, C20:4, C16:0, C18:0, C18:1) increased in old-age quadriceps muscle. In contrast, levels of most of these free fatty acids were reduced in old-age BAT (Fig.4c and **Extended Data Fig. 4b)**, indicating diminished lipolysis in BAT and a reduced pool of free fatty acid activators of UCP1^26^. Similar to free fatty acids, we also discovered contrasting changes in acylcarnitines between BAT and quadriceps muscle tissues (Fig.4d). Acylcarnitines are the shuttles of fatty acids into and out of mitochondria, reflecting the use of acyl-CoAs in beta-oxidation and preventing any build-up of CoA levels in the mitochondrial matrix. Notably, levels of LC- and VLC-carrying acylcarnitines increased in quadriceps muscle of old-aged mice, whereas BAT showed no systematic accumulation of LC- or VLC- acylcarnitines. Instead, levels of several short-chain acylcarnitines decreased, indicative of a reduced oxidation of branched chain amino acid (BCAA). Several other tissues showed similar accumulation of free fatty acids and acylcarnitines as noted for quadriceps muscle **(Extended Data Fig.5a and 5b),** for example, in heart tissues. Overall, the concerted increase in levels of free fatty acids and acylcarnitines supports a system-level redistribution of lipid oxidation towards muscle and other non-fat tissues, in contrast to diminished use of beta-oxidation in BAT mitochondria.

### Shifts in thermogenic signaling and the cost of compensation

**Figure 5:**
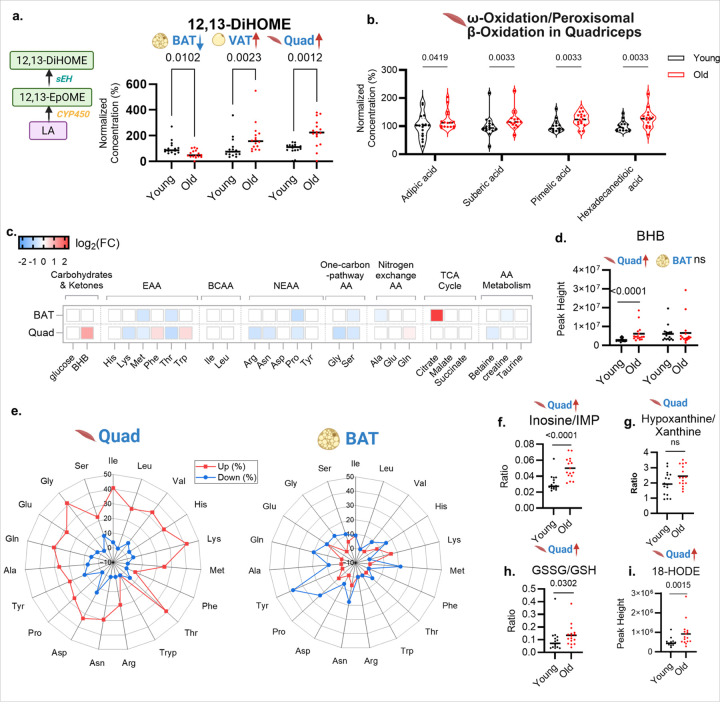
Thermogenic signal shifts in BAT versus quadriceps muscle and metabolic consequences in old-age mice. **(a)** Pathway for linoleate (LA)-derived oxylipins to 12,13-DiHOME and tissue levels of 12,13-DiHOME between young and old mice in BAT, VAT and quadricep muscle. Dots: individual animals; horizontal lines: median; q values: FDR-adjusted p-values. **(b)** Violin plots of dicarboxylic acids levels in quadricep muscle of young and old mice. Dots: individual animals; horizontal lines: median; q values: FDR-adjusted p-values. **(c)** Heat map of log2-fold changes (FC) between old and young mice for carbohydrate, ketone, and amino acids and TCA cycle. EAA: essential amino acid; BCAA: branched chain amino acid; NEAA: non-essential amino acid; AA: amino acid (raw p<0.05). **×** denotes not detected. **(d)** β-hydroxybutyrate (BHB) levels in quadriceps muscle and BAT of young and old mice. q values: FDR-adjusted p-values. **(e)** Amino acid residue-level radar plots for peptide regulation in quadriceps muscle (left) and BAT (right). For each amino acid residue, values (%) indicate the proportion of detected di/tri-peptides containing that residue that were significantly up- or down-regulated (raw p<0.05). (f–i) Oxidative stress biomarkers levels in quadriceps muscle of young and old mice. **(f)** inosine/inosine monophosphate (IMP) ratio; **(g)** hypoxanthine/xanthine ratio; **(h**)oxidized glutathione/glutathione ratio (GSSG/GSH); **(i)** 18-hydroxylinoleic acid (18-HODE) levels. Dots: individual animals; horizontal lines: median; q values: FDR-adjusted *p-*values.

Given diminished participation of BAT in lipid oxidation at old age, concomitant with increased use of lipids in skeletal muscle, we evaluated the regulation of the thermogenic mediator 12,13-DiHOME^23^. At old age, 12,13-DiHOME levels decreased in BAT and increased in VAT and quadriceps (Fig.5a), consistent with weakened BAT thermogenic signaling and a shift of the adaptive thermogenic workload to peripheral oxidative tissues, such as VAT and quadriceps. The corresponding accumulation of dicarboxylic acids (adipic, suberic, pimelic, and hexadecanedioic acid) in quadriceps muscles of old-age mice (Fig.5b) reveals a shift toward ω- and peroxisomal β-oxidation, a compensatory response to mitigate fatty acid burden when mitochondrial β-oxidation capacity is exceeded^27, 28^. We then examined 26 abundant metabolites (amino acid metabolism, TCA cycle, glucose and β-hydroxybutyrate) that serve as building blocks and energy sources^29^ and usually carry high metabolic flux (Fig.5c–d). BAT of old mice showed minimal metabolic remodeling, restricted to citric acid (5-fold increase, indicating its export from mitochondria under stress conditions) and 6/21 compounds in amino acid metabolism. In contrast, quadriceps muscle of old age mice exhibited a pronounced increase of β-hydroxybutyrate levels (β-HB, FC=2.1; q-value <0.0001), indicating an increased use of lipid-derived ketone bodies as energy fuel. Concurrently, amino acid metabolism showed drastic changes in quadriceps muscle in old versus young mice, with 12/21 compounds found to be significantly different. While most amino acids were downregulated in old mice, two aromatic (essential) amino acids were found at 30% increased levels in quadriceps of old mice (phenylalanine and tryptophan). In contrast, citric acid was not upregulated in quadriceps muscle, indicating a functionally normal TCA cycle. Similar to amino acids, BAT and quadriceps muscle also showed significant differences in the abundance of small peptides that mostly indicate proteolysis. Tissue-specific metabolic reprogramming was further underscored by the analysis of small peptides levels (Fig.5e). In quadriceps of old mice, di- and tri-peptides were broadly upregulated. When virtually digesting these peptides into amino acid residues, we consistently found about 25–40% of all upregulated peptides to be constituted by all amino acids. This pattern is consistent with elevated proteolytic activity in skeletal muscle in old mice. Yet, for the amino acids tryptophan and phenylalanine we found that peptides that contained these two amino acids were very rarely found to be either up- or downregulated (Fig.5e). On the other hand, these two amino acid-residues were also found to be upregulated as free amino acids in quadriceps muscle (Fig. 5c), suggesting selective degradation or rapid turnover of peptides harboring these residues. In combination, this observation supports the concept of general protein breakdown in muscle of old-age mice, with even increased and specific hydrolysis of Trp- and Phe-containing peptides into free amino acids. Conversely, BAT from old mice exhibited many more down-regulated di- and tri-peptide levels, particularly those containing Tyr, Pro, Met and Gln amino acid residues, with proportions >20% (Fig.5e). Hence, unlike quadriceps muscle, protein hydrolysis into peptides was decreased in BAT, consistent with a switch in thermogenic use and divergent rewiring in skeletal muscle versus BAT.

Nonetheless, the compensatory shift in fuel use in skeletal muscle of old-age mice, including protein degradation, incurs a substantial metabolic burden. The elevated inosine-to-IMP ratio (Fig.5f) is consistent with enhanced purine catabolism^30^, concordant with downstream metabolites that showed an increased trend (but not significant) in hypoxanthine/xanthine ratio (Fig.5g)^30^. This process coincides with a pronounced redox imbalance, marked by elevated oxidized glutathione/glutathione (GSSG/GSH) ratio (Fig.5h)^31^ and increased levels of the lipid peroxidation products 18-hydroxylinoleic acid (18-HODE, Fig.5i)^32^ at old-age mice muscles. Overall, our data depict the quadriceps muscle as a tissue under duress in old age, where sustained lipid oxidation imposes energetic strain and oxidative stress. We propose that age-related loss of thermogenesis in BAT diverts thermogenic demand to other organs such as skeletal muscle. In turn, muscles at old age compensate via heightened oxidative metabolism at the cost of proteolytic activation and redox stress, contributing to functional decline.

## Discussion

While we present a multi-tissue metabolomics atlas of 2,875 annotated metabolites, even more features detected by LC-MS/MS remained structurally unassigned. We provide all metabolic annotations in a public, online repository for scientific review and critique. Continued curation with authentic standards, expanded MS/MS libraries, and improved *in-silico* matching will refine identifications and pathway mapping lead to further extended atlases in the future. Yet, the current suite of annotated compounds documented here revealed that old-age reconfigures systemic energy balance through coordinated, organ-specific remodeling of fuel metabolism and cardiolipin-inferred regulation of composition of the inner mitochondrial membrane. Specifically, thermogenesis-relevant metabolites converge on a common pattern in aging: as BAT loses mitochondrial capacity, skeletal muscle assumes a greater lipid-oxidation workload, with substantial energetic and redox costs. Prior work has shown reduced BAT activity and increased muscle sarcopenia with age^10, 11^. Our data extend these observations by resolving organ-specific biochemical changes that link thermogenic control at old-age with substrate availability, lipidmediator signaling, and changes in mitochondria cristae inferred from cardiolipin profiles. We found that a central signature of lipid aging was the synthesis of cardiolipins within the inner mitochondrial membrane. Nascent cardiolipins decline across multiple organs (e.g., liver, intestinal tissues) at old-age, most prominently in BAT, whereas heart and skeletal muscle remained largely intact, though some linoleate-rich mature cardiolipins are selectively downregulated in heart tissues. These patterns are consistent with ultrastructure imaging in aging mice: 3D electron microscopy shows a rarefaction of cristae in BAT^33^, whereas heart mitochondria are maintained^34^. Liver and kidney exhibit age-related membrane changes including apoptosis-like subpopulations^34^. Given the critical role of cardiolipins in stabilizing the curvature of cristae and mitochondrial respiratory supercomplexes^35^, these observations suggest corresponding mitochondrial dysfunction in late life. This dysfunction leads to diminished BAT output for thermogenesis and necessitates fuel reallocation toward peripheral oxidative tissues, including quadriceps muscle.

Because the biosynthesis and maturation of cardiolipin is required for uncoupling lipid beta-oxidation from ATP generation in BAT^19^, we analyzed the data whether BAT displays a concurrent metabolite-level loss of thermogenic readiness. Previously, short- and medium-chain triacylglycerides (TGs) were defined as more readily mobilizable for beta-oxidation than long- or very long TGs^20, 21^. Correspondingly, we found the same pattern in BAT of old-age mice, consisting of a decline of short- and medium-chain TGs while the total amount of TG remained unchanged. Similarly, we found a broad decrease in levels of long- and very-long chain free fatty acids in BAT, indicating diminished lipolysis and a reduced pool of free fatty acid that act as activators for uncoupling protein 1 (UCP1)^26^. Concurrently, these free fatty acids as well as the corresponding acylcarnitines were found to be increased in both plasma and in quadriceps muscle, demonstrating a shift in fuel utilization across organs. In BAT, levels of long- and very-long chain acylcarnitines did not change at old age, suggesting that mitochondrial transport within BAT is unlikely to be the primary bottleneck of the thermogenic shifts in fuel utilization at old age.

The decline of 12,13-DiHOME levels in BAT is particular informative, indicating compromised thermogenic signaling^23^. Circulating 12,13-DiHOME has been reported to decline with age in humans and rodents^36^. BAT is a principal source of 12,13-DiHOME during cold or exercise, and this lipokine acutely enhances fatty acid uptake and oxidation in both BAT and skeletal muscle by promoting FATP1 and CD36 translocation^23, 37^. In our metabolome atlas, a tissue-divergent pattern was observed between young and old mice: 12,13-DiHOME decreased in BAT but increased in VAT and quadriceps, consistent with diminished BAT output together with compensatory production and preferential targeting in peripheral tissues that increasingly shoulder the β-oxidation burden.

Skeletal muscle adapts to aging by adopting a high-turnover lipid phenotype. In aging quadriceps muscle, levels of long- and very-long chain fatty acids and acylcarnitines increased across chain-length classes. Similarly, bulk levels of mono- and diacylglycerides increased while bulk TG remained unchanged at old age, accompanied by remodeling of the TG pool toward longer, more unsaturated species and depletion of shorter saturated species^20, 21^. In the quadriceps of old mice, auxiliary fatty acid oxidation pathways appears increasingly engaged, specifically ω-oxidation in the endoplasmic reticulum and peroxisomal β-oxidation pathways, as evidenced by elevated levels of dicarboxylic acids that mark compensatory when mitochondrial β-oxidation is heavily loaded^27, 28^. Despite this high oxidative workload, muscle mitochondria remained functional at old age, with inner mitochondrial membrane integrity relatively preserved and stable citrate levels, supporting sustained respiration. These metabolic signatures align with transcriptomics data of human skeletal-muscle showing age-related remodeling of oxidative phosphorylation and lipid metabolism^38^.

However, this adaptation of muscle metabolism at old age comes with costs. We found increased inosine/IMP ratios that indicate metabolic shifts toward purine breakdown, higher ratios of oxidative stress markers GSSG/GSH and increased levels of 18-HODE increase, and accumulation of small peptides indicating protein hydrolysis. All these shifts indicate redox stress with accelerated protein turnover^39^. The resulting proteolysis and amino acid diversion to support anaplerosis and antioxidant buffering may compromise contractile protein maintenance^39^, providing a biochemical context for sarcopenia and reduced muscle performance in aging. This metabolic phenotype of stress-and-turnover suggests that interventions which could lower oxidative load and support mitochondrial function might help preserve muscle quality and thermogenic capacity in late life.

In summary, we find that thermogenesis is reprogrammed at old age at the organ level: lipid remodeling in the inner mitochondrial membrane accompanies attenuation of BAT thermogenesis while the skeletal muscle assumes a larger share of lipid oxidation. This redistribution imposes metabolic strains and helps explain reduced cold defense in older adults. In BAT and skeletal muscle of old mice, we observed no dominant differences between male and female, while several other organs displayed greater sexual dimorphism. Future work may detail further metabolic adaptations with aging. For example, to quantify flux partitioning between organs and organelles, stable-isotope tracing with arteriovenous sampling or tracer clamp studies will be required. Tissue resolution can be extended by profiling distinct muscle types with divergent fiber composition (for example, soleus, gastrocnemius, extensor digitorum longus). Together, these steps will evolve the atlas from a static map into a dynamic framework for tracking and modulating metabolic aging across organs.

## Methods

### Tissue Collection

C57BL/6N-Crl from the Mouse Biology Program (MBP) B6N colony were aged to either 16 or 90–96 weeks of age and euthanized for tissue collection. Mice housing was in accordance with protocol approved by the UCD Institutional Animal Care and Use Committee (IACUC) and with the Association for Assessment and Accreditation of Laboratory Animal Care, International (AAALAC). Mice were multi-housed in ventilated cages (Optimice IVC, Animal Care Systems, Centennial, CO) on standardized environmental conditions (12:12 hour (06:00–18:00) light cycle during; room temperature 20–22°C). Mice were given free access to water and standard laboratory rodent chow (Rodent chow, Teklad 2918). For tissue collection, mice were anesthetized with isoflurane and perfused with ice cold PBS (around 40 ml) through the heart left ventricle. Urine (n = 11 per age group) and cerebrospinal fluid (CSF; n= 8 old, n = 16 young) were collected from a subset of mice. Hippocampus, heart, lungs, spleen, thymus, pancreas, gallbladder, stomach, duodenum, jejunum, ileum, colon and cecum, brown, subcutaneous and visceral adipose tissue, quadriceps muscle, kidney, bladder, testes and uterus, liver, feces and plasma were collected from n= 8 males and 8 females at 16 and 90–96 weeks, flash frozen and stored at −80°C until analyzed, yielding a total of 783 samples. Detailed individual animal metadata were given in Supplementary Table 3.

### Sample preparation

Briefly, ten milligrams of tissue were homogenized with three 3 mm metal beads added in 225 μl of −20°C cold, internal standard-containing methanol using a GenoGrinder 2010 (SPEX SamplePrep) for 1 min at 1,350 rpm. Biofluid samples were thawed, vortex and aliquot 20 μl of urine and plasma or 2 μl of CSF and transferred in 2 ml Eppendorf tube and then mixed with internal standard containing ice-cold methanol. The extraction methanol contained the following internal standards for quality control and retention time normalization: sphingosine (d17:1), LPE (17:1), LPC (17:0), MG (17:0/0:0/0:0), DG (12:0/12:0/0:0), PC (12:0/13:0), cholesterol-d_7_, SM (18:1/17:1), ceramide (d18:1/17:0), PE (17:0/17:0), TG (14:0/16:1/14:0)-d_5_, TG (17:0/17:1/17:0)-d_5_, acylcarnitine (18:1)-d_3_, fatty acid (16:0)-d_3_, MAG (17:0/0:0/0:0), PI (15:0–18:1)-d_7_, PG (17:0/17:0), PS (15:0–18:1)-d_7_, glucosylceramide(d18:1/17:0), mono-sulfo galactosylceramide (d18:1/17:0), and 5-PAHSA-d_9_. The homogenate was vortexed for 10 s. 750 μL of −20 °C cold, internal standard-containing methyl tertiary-butyl ether (MTBE) was added, and the mixture was vortexed for 10 s and shaken at 4 °C for 5 min with an Orbital Mixing Chilling/Heating Plate (Torrey Pines Scientific Instruments). MTBE contained cholesteryl ester 22:1 as internal standard. Next, 188 μL room temperature water was added and vortexed for 20 s to induce phase separation. After centrifugation for 2 min at 14,000**×***g*, two 350 μL aliquots of the upper non-polar phase and two 125 μL aliquots of the bottom polar phase were collected and dried down by SpeedVac (Labconco). Two types of QC pools were prepared, the mega QC was prepared by remaining fractions from each samples and were injected after every 10 biological samples. Tissue-specific QC pools (26 types of tissues) were prepared by the remaining fractions of 32 samples per tissue type for iterative MS/MS exclusions.

The non-polar phase employed for lipidomics was resuspended in a mixture of methanol/toluene (60 μL, 9:1, v/v) containing an internal standard [12-[(cyclohexylamine) carbonyl]amino]-dodecanoic acid (CUDA)] before injection. Resuspension of dried polar phases for HILIC analysis was performed in a mixture of acetonitrile/water (90 μL, 4:1, v/v) containing the following internal standards: CUDA, caffeined_9_, acetylcholine-d_4_, TMAO-d_9_, 1-methylnicotinamide-d_3_, Val-Tyr-Val, betaine-d_9_, acyl carnitine(2:0)-d_3_, N-methyl-histamine-d_3_, l-carnitine-d_3_, butyrobetaine-d_9_, l-glutamine-d_5_, aspartic acid-d_3_, l-arginine-^15^N_2_, cystine-d4, asparagine-d3, histidine-d5, isoleucine-d10, leucine-d10, methionine-d8, ornithine-d2, phenylalanine-d_8_, proline-d_7_, threonine-d_5_, tryptohan-d_8_, tyrosine-d_7_, valine-d_8_, spermine-d_8_, glucose-d_7_, fructose-6-phosphate-^13^C_6_, succinic acid-d_4_, taurocholic acid-d_4_, adenosine 5′-monophosphate-^15^N_5_, uridine 5′-monophosphate-^15^N_2_, dopamine-d_4_, taurine-d_4_, uracil-d_2_, biotin-d_4_, N-acetylalanine-d_3_, guanine-^13^C, and adenosine-^13^C_5_.

### Lipidomic LC-MS/MS analysis

Thermo Scientific Orbitrap Exploris 240coupled with Vanquish UHPLC system (Thermo Scientific, Waltham, MA, USA) was used for RPLC-MS/MS analysis with the focus on nonpolar lipids. RP separation was performed on a Waters ACQUITY Premier BEH C18 column (50 mm **×** 2.1 mm; 1.7 μm) with the VanGuard FIT guard column (Waters Corporation, Milford, MA, USA). The column was maintained at 65 °C with flow rate of 0.8 mL/min. For ESI positive ion mode, mobile phase A was acetonitrile/water (60/40, *v/v*) with 0.1% formic acid and 10 mM ammonium formate, and mobile phase B was 2-propanol/acetonitrile (90:10, v/v) with 0.1% formic acid and 10 mM ammonium formate. For ESI negative ion mode, mobile phase A was acetonitrile/water (60/40, v/v) with 10 mM ammonium acetate, and mobile phase B was 2-propanol/acetonitrile (90/10, v/v) with 10 mM ammonium acetate. Same elution gradient was used for both ion modes: 0 min: 15% B; 0.75 min: 30% B; 0.975 min: 48% B; 4 min, 82% B; 4.125 min, 99% B; 4.5 min, 99% B; 4.58 min, 15% B; 5.5 min, 15% B. Sample injection volume was 3 μL for positive and 5 μL for negative mode. The Orbitrap MS was operated in data-dependent acquisition mode, under the following conditions: spray voltage, 3.5 kV; ion transfer tube temperature, 320 °C; sheath gas flow rate, 60; auxiliary gas flow rate, 18; sweep gas flow rate, 1; vaporizer temperature, 290 °C. The following acquisition parameters were used for MS1 analysis: resolution, 60,000; scan range, 120–1,700 m/z; time, 100 ms; RF lens, 80%, spectrum data type, centroid. Data-dependent MS/MS parameters: resolution, 15,000; times, 50 ms; normalized AGC target (%), 100; RF lens (%), 70; loop count, 2; TopN, 2; isolation window, 1.0 m/z; HCD collision energies (%) 20, 30, 40; spectrum data type, centroid; other parameters were custom unless stated. To improve MS/MS coverage, four runs with iterative MS/MS exclusions were performed using *Acquire X* (Thermo Scientific, Waltham, MA, USA) for both mega pool QC and tissue-specific pooled QC (26 types of tissues) in positive and negative electrospray conditions to avoid repeated acquisition of MS/MS spectra. Samples were acquired in the sequence randomized per type of tissues.

### HILIC-MS/MS analysis

For LC-MS/MS analysis of polar metabolites, Thermo Scientific Q Exactive HF-X MS Quadrupole Orbitrap MS System coupled with Vanquish UHPLC system (Thermo Scientific, Waltham, MA, USA). A Waters ACQUITY Premier BEH Amide VanGuard FIT column (50 mm **×** 2.1 mm i.d., 1.7 μm) was used for separation of polar metabolites. Sample injection volume was 3 μl in positive and 5 μl for negative mode. Mobile phase A consisted of water with 10 mM ammonium formate and 0.125% formic acid, and mobile phase B consisted of acetonitrile/water (95/5, v/v) with 10 mM ammonium formate and 0.125% formic acid. The gradient, including re-equilibration, was as follows: 0 min, 100% B; 0.5 min, 100% B; 1.95 min, 70% B; 2.55 min, 30% B; 3.15 min, 100% B; 3.8 min, 100% B. The following acquisition parameters were used for MS1 analysis: resolution, 60,000; AGC target, 1e6; maximum IT, 100 ms; scan range, 60–900 m/z; data type, centroid. The following acquisition parameters were used for MS/MS analysis: resolution, 15,000; AGC target, 1e5; maximum IT, 50 ms; loop count, 2; ToPN, 2; isolation window, 1.0 m/z; scan range: 200–2,000 m/z; fixed first mass, 50.0 m/z; (N)CE/Stepped (N)CE, 30, 50, 80; data type, centroid; minimum AGC target, 8.00e3; intensity threshold, 1.6e5; exclude isotopes, on; dynamic exclusion, 2.0s. The Orbitrap MS was operated in data-dependent acquisition mode, under the following conditions: spray voltage, 2.5 kV (negative mode) and 3.5 kV (positive mode), capillary temperature 250 °C; sheath gas, 55; auxiliary gas, 17.5; spare gas 4; probe heater temperature, 420 °C; S-Lens RF level, 60. Mass spectrometer parameters for both positive and negative ion modes were set as follows: resolution, 60,000; AGC target 1e6; maximum IT 100 ms, scan range: 60 to 900 m/z, spectrum data type, centroid; MS/MS acquisition method: data-dependent acquisition mode; resolution, 15,000; AGC target, 10e5; maximum IT, 50 ms, isolation window: 1.0 m/z; scan range, 200 to 2000 m/z, stepped collision energies: 30, 50, 80, minimum AGC target 8e3, intensity threshold 1.6 e5. dynamic exclusion, 2.0s. To increase the total number of MS/MS spectra, four runs with iterative MS/MS exclusions were performed using the R package “IE-Omics” for both mega pool QC and tissue-specific pooled QC in positive and negative mode. Samples were acquired in the sequence randomized per type of tissues.

### LC-MS data processing and statistics

Raw MS data files were converted to mzML format using ProteoWizard MSConvert (ver. 3.0). The converted data were processed by MassCube^40^ (https://github.com/huaxuyu/masscube) combined with Mass.Wiki (https://masswiki.metabolomics.us). MassCube was used for feature detection, chromatographic peak evaluation, isotope/adduct/in-source fragment grouping, with metabolite annotation. A msp. files containing all MS/MS will be automatically generated and was further uploaded to Mass.Wiki “Analyze Multiple Spectra” function to automatically search all available libraries. In parallel, these data were also automatic data processed by the LC-BinBase database environment that utilizes raw files including MS1 and MS/MS spectra through the cloud-enabled peak finding and data processing code of MS-DIAL software vs. 4.90^41^. MassCube parameters were: MS1 intensity cutoff, 500; MS2 intensity cutoff, 200; m/z tolerance, 10 mDa; scan-to-scan correlation threshold for feature grouping, 0.7; retention time tolerance for alignment: 0.2 minute; gap filling by forced local maximum detection: enabled; automatic retention time correction: enabled; quantification method: peak height; MS signal normalization algorithm, LOWESS; automatic batch recognition, enabled. In Mass.Wiki, the combined MS/MS spectra were searched against all world-wide available mass spectral repositories, including databases MassBank of North America (https://massbank.us), MassBank of Europe (https://massbank.eu), MassBank of Japan (https://massbank.jp), the MS-DIAL metabolomics MSP spectral kit, lipid blast libraries (https://systemsomicslab.github.io/compms/msdia), GNPS (https://gnps.ucsd.edu), NIST 23 (https://chemdata.nist.gov/) and the Agilent METLIN Metabolomics database. MS/MS spectral matching used unweighted entropy similarity with precursor ion removal enabled. Similarity cutoffs were 0.7 for matches to standard-confirmed libraries and 0.5 for *in silico* predicted libraries. In case where structurally diagnostic fragment ions were present, a lower similarity threshold was permitted. All annotations for the Aging Mouse Atlas presented here are publicly available at MassWiki with the permanent URLs (HILIC positive: https://masswikilab.metabolomics.us/experiment/aBXKFUA?isPublic=true; HILIC negative: https://masswikilab.metabolomics.us/experiment/a3APPSC?isPublic=true; Lipidomics positive: https://masswikilab.metabolomics.us/experiment/a21BEJV?isPublic=true; Lipidomics negative: https://masswikilab.metabolomics.us/experiment/aJZ2QVA?isPublic=true). The Aging Mouse Atlas represents the most comprehensive aging mouse organ-resolved metabolome published so far, including MSI-compliant confidence levels and retention time prediction^42, 43^. We defined metabolites into 18 chemical superclasses using the RefMet classification system^12^. Systematic error removal was performed by random forest (SERRF software, https://slfan2013.github.io/SERRF-online/#)^44^. For metabolites that were detected by two or more platforms, values with the lowest relative standard deviation in quality control samples were kept. Metabolites that were presented in at least 28 of the 32 samples in at least one of the tissue type groups were kept in the dataset, otherwise, metabolites were removed from the dataset. Missing data were replaced by 1/10th of the minimal value (default value 100).

Statistical analysis was performed by normalization to the median intensity of all identified compounds, log transformation, and Pareto scaling. UMAP analysis in R was used for multivariate statistics and visualization, specifically for outlier detection. For group comparison (old vs. young), two-sided Mann-Whitney U tests were applied using GraphPad Prism (version 10). To summarize aging effects per organ, we use raw *p* < 0.05 counts as a descriptive metric (no interference). All single metabolite conclusions use BH-FDR-adjusted *q*-values. For each metabolite within a given tissue, intensities were analyzed by two-way ANOVA with fixed factors age (young, old), sex (female, male), and their interaction (age **×** sex). P values for the age, sex, and age **×** sex terms were computed from the ANOVA table. Multiple testing was controlled within tissue across all metabolites using the Benjamini–Hochberg procedure; features with FDR < 0.05 were considered significant. All analyses were performed in R.

### Visualization of results

Graphs were made using GraphPad Prism (version 10) and RAWGraphs 2.0 (https://app.rawgraphs.io/). Statistical analysis was performed with GraphPad Prism (version 10). Figures Fig.1a & 1c, Fig 2a & 2b, Fig 3a, 3e & 3f, Fig.4a & 4b and Fig. 5a & 5b were created in https://BioRender.com.

## Supplementary Files

This is a list of supplementary files associated with this preprint. Click to download.

- ExtendedDataFigure.docx
- SupplementaryInformationAgingMiceAtlasFinal.docx

## Data Availability

Raw MS data from this study are available on GNPS MassIVE under accession number MSV000099388 [Lipidomics: ftp://massive-ftp.ucsd.edu/v11/MSV000099388/] and MSV000099387 [HILIC: ftp://massive-ftp.ucsd.edu/v11/MSV000099387/]. Sample IDs individual animal metadata were provided in Supplementary Table 3. Experimental MS data for all annotated metabolites were provided in the Supplementary Table 4 the Mass.Wiki platform (HILIC positive: https://masswikilab.metabolomics.us/experiment/aBXKFUA?isPublic=true; HILIC negative: https://masswikilab.metabolomics.us/experiment/a3APPSC?isPublic=true; lipidomics positive: https://masswikilab.metabolomics.us/experiment/a21BEJV?isPublic=true; lipidomics negative: https://masswikilab.metabolomics.us/experiment/aJZ2QVA?isPublic=true). For metabolite annotation, the commercial NIST23 Tandem Mass Spectral Library used in biological applications can bea purchased from NIST [https://www.nist.gov/programs-projects/nist23-updates-nist-tandem-and-electron-ionization-spectral-libraries]. The commercial Agilent METLIN Metabolomics database can be purchased from Agilent [https://www.agilent.com]. MassBank of North America (MoNA) database can be downloaded from MoNA [https://massbank.us]. MS-DIAL metabolomics MSP spectral kit can be downloaded from the MS-DIAL website [https://systemsomicslab.github.io/compms/msdial/main.html#MSP], and GNPS Library can be downloaded from the GNPS website [https://external.gnps2.org/gnpslibrary]. The Metabolome Atlas is openly available at our GitHub repository [https://github.com/minliuUCDavis/AgingMiceAtlas]. An interactive web tool will be released soon to enable easy public access and exploration.
